# Long-term effect of critical illness after severe paediatric burn injury on cardiac function in adolescent survivors: an observational study

**DOI:** 10.1016/S2352-4642(17)30122-0

**Published:** 2017-10-20

**Authors:** Gabriel Hundeshagen, David N Herndon, Robert P Clayton, Paul Wurzer, Alexis McQuitty, Kristofer Jennings, Ludwik Branski, Vanessa N Collins, Nicole Ribeiro Marques, Celeste C Finnerty, Oscar E Suman, Michael P Kinsky

**Affiliations:** 1 Department of Surgery, University of Texas Medical Branch, 301 University Blvd, Galveston, TX 77555; 2 Shriners Hospitals for Children, Galveston, 815 Market St, Galveston, TX 77555; 3 Department of Hand, Plastic and Reconstructive Surgery, Burn Trauma Center, BG Trauma Center Ludwigshafen; University of Heidelberg, Ludwig-Guttmann-Str. 13, 67071 Ludwigshafen, Germany; 4 Division of Plastic, Aesthetic and Reconstructive Surgery, Department of Surgery, Medical University of Graz, Austria; 5 Department of Anesthesiology, University of Texas Medical Branch, 301 University Blvd, Galveston, TX 77555; 6 Office of Biostatistics, Department of Preventive Medicine and Community Health, University of Texas Medical Branch, 301 University Blvd, Galveston, TX 77555

## Abstract

**Background:**

Sepsis, trauma, and burn injury acutely depress systolic and diastolic cardiac function; data on long-term cardiac sequelae of pediatric critical illness are sparse. This study evaluated long-term systolic and diastolic function, myocardial fibrosis, and exercise tolerance in survivors of severe pediatric burn injury.

**Methods:**

Subjects at least 5 years after severe burn (post-burn:PB) and age-matched healthy controls (HC) underwent echocardiography to quantify systolic function (ejection fraction[EF%]), diastolic function (E/e′), and myocardial fibrosis (calibrated integrated backscatter) of the left ventricle. Exercise tolerance was quantified by oxygen consumption (VO_2_) and heart rate at rest and peak exercise. Demographic information, clinical data, and biomarker expression were used to predict long-term cardiac dysfunction and fibrosis.

**Findings:**

Sixty-five subjects (PB:40;HC:25) were evaluated. At study date, PB subjects were 19±5 years, were at 12±4 years postburn, and had burns over 59±19% of total body surface area, sustained at 8±5 years of age. The PB group had lower EF% (PB:52±9%;HC:61±6%; p=0.004), E/e′ (PB:9.8±2.9;HC: 5.4±0.9;p<0.0001), VO_2peak_ (PB:37.9±12;HC: 46±8.32 ml/min/kg; p=0.029), and peak heart rate (PB:161±26;HC:182±13bpm;p=0.007). The PB group had moderate (28%) or severe (15%) systolic dysfunction, moderate (50%) or severe diastolic dysfunction (21%), and myocardial fibrosis (18%). Biomarkers and clinical parameters predicted myocardial fibrosis, systolic dysfunction, and diastolic dysfunction.

**Interpretation:**

Severe pediatric burn injury may have lasting impact on cardiac function into young adulthood and is associated with myocardial fibrosis and reduced exercise tolerance. Given the strong predictive value of systolic and diastolic dysfunction, these patients might be at increased risk for early heart failure, associated morbidity, and mortality.

**Funding:**

Conflicts of Interest and Sources of Funding: The authors do not have any conflicts of interest to declare. This work was supported by NIH (P50 GM060338, R01 GM056687, R01 HD049471, R01 GM112936, R01-GM56687 and T32 GM008256), NIDILRR (H133A120091, 90DP00430100), Shriners Hospitals for Children (84080, 79141, 79135, 71009, 80100, 71008, 87300 and 71000), FAER (MRTG CON14876), and the Department of Defense (W81XWH-14-2-0162 and W81XWH1420162). It was also made possible with the support of UTMB’s Institute for Translational Sciences, supported in part by a Clinical and Translational Science Award (UL1TR000071) from the National Center for Advancing Translational Sciences (NIH).

## Introduction

Whenever a child is discharged from intensive care, the immediate feeling of relief for having averted a life-threatening crisis naturally outweighs concerns over longstanding consequences. There is evidence, that pediatric critical illness due to trauma, sepsis, or burn injury induces considerable systemic perturbations and stress through severe inflammation, hypermetabolism, and a protracted surge of serum catecholamines, that can persist for years with little known aftereffects.^1–3^ Advances in critical care emphasize the importance of studying long-term sequelae of critical illness, including cognitive ability, quality of life, and functional status.^4,5^ The long-term cardiovascular outcomes of critical illness remain largely unknown.

While ample evidence suggests that cardiac dysfunction can occur in critically ill patients, there are limited and inconclusive data regarding long-term cardiac function in survivors of pediatric critical illness, with this data being derived mainly from studies of small and diverse study populations.^6^ A major challenge in conducting prospective long-term studies of cardiac outcomes in pediatric sepsis and trauma is the significant loss to follow-up that occurs once the underlying condition is resolved and patients are discharged.^6^ The treatment of severe pediatric burn injury, which induces systemic effects comparable to those of severe trauma and sepsis,^2^ is unique at our institution and other specialized pediatric burn centers in that patients continuously return for reconstructive and rehabilitative procedures long after discharge from critical care. Thus, this particular patient population may provide insights into long-term cardiac function after pediatric critical illness.

Basic science studies show that acute systolic and diastolic dysfunction after burn injury result from circulating depressants such as pro-inflammatory cytokines (eg, tumor necrosis factor alpha [TNFα] and interleukin 1 beta [IL-1β]), gut-derived factors from plasma and mesenteric lymphatics, and other neurohumoral mediators.^7^ Two recent population-based longitudinal studies were conducted in adults who sustained burn injuries as children or young adults to assess long-term morbidity and mortality due to a variety of cardiovascular diagnoses.^8,9^ Although the spectrum of cardiovascular diagnoses included in these analyses was broad, the authors reported significant increases in incidence and length of hospitalization as well as mortality related to cardiovascular disease among middle-aged survivors of pediatric burn injury. The importance of this clinical problem may be considerable given that systolic and diastolic dysfunction are powerful predictors of poor cardiovascular prognosis, morbidity, and mortality in young adults.^10^

The purpose of this study was to determine long-term cardiac function and exercise capacity in pediatric burn survivors. Clinical and pathophysiological factors contributing to systolic and diastolic dysfunction as well as myocardial fibrosis were also evaluated. The collection of pilot data from this trial could serve as a template for the evaluation of long-term cardiac sequelae in other types of pediatric critical illness.

## Methods

### Study design and participants

The institutional review board of the University of Texas Medical Branch, Galveston, TX, approved this study and informed consent was obtained prior to enrollment of each subject. Between 2016 and mid-2017, we prospectively studied 40 consecutive subjects returning to our institution for long-term follow-up or reconstructive procedures, who had a history of severe pediatric burn injury affecting at least 30% of the total body surface area (TBSA), sustained the injury at least 5 years prior to enrollment, and were treated acutely at Shriners Hospitals for Children – Galveston (Galveston, TX). The control group consisted of a convenience sample of 25 healthy volunteers who underwent echocardiographic evaluation for systolic and diastolic function as well as exercise testing during the same time period.

### Demographic and medical data

Collected data included demographics (age, sex, age at burn, dates of burn and admission, burn size, burn depth, and mechanism of injury), information concerning acute hospitalization (delay of admission [DA], length of hospitalization, days of mechanical ventilation, total number of operations), concomitant injuries (inhalation trauma, sepsis), Baux – score (burn size in %TBSA + 17, if inhalation injury present) and receipt of research medication (propranolol, oxandrolone, placebo, other). Body mass index (BMI) at the time of echocardiographic and exercise assessments was calculated for the study groups as [BMI = mass (kg)/height^2^ (m)]. The study subjects’ personal and relevant family medical history were recorded; all subjects were screened for congenital heart disease, which was an exclusion criterion for this study.

### Outcome measures

#### Systolic and diastolic function

Study participants underwent transthoracic two-dimensional echocardiography, which was performed by one experienced echocardiographer using GE Vivid 9 pro (Milwaukee, WI). Ejection fraction is a readily available and reliable parameter of systolic function^11^: end-diastolic volume (EDV) and end-systolic volume (ESV) were determined using modified Simpson’s rule from a two-dimensional tracing of the left ventricular (LV) area and length in the parasternal LV long axis for transthoracic echocardiography during end-expiration of 3 to 5 representative cardiac cycles. Ejection Fraction (EF%) was calculated as EF% = (EDV-ESV)/EDV*100, recorded, and classified as normal (EF% > 50), moderate dysfunction (EF% = 50-41), or severe dysfunction (EF% ≤ 40).^10^

Diastolic function was obtained from representative recordings over 3 to 5 cardiac cycles at end-expiration. Pulsed-wave Doppler was interrogated across the mitral valve. Early and late peak mitral inflow velocities (E and A waves, respectively) were recorded. Tissue Doppler imaging was then performed by placing the Doppler cursor within 1 cm of the lateral insertion point of the mitral leaflets to determine the longitudinal excursion of the mitral annulus during diastole in order to record early diastolic ventricular velocity (e′). Ventricular compliance was assessed by ratios of trans-mitral E to A velocity (E/A) and the ratio of E velocity to e′ (E/e′). E/e′ is a measure of diastolic function that is independent of hemodynamic confounders such as tachycardia or preload.^11,12^ Subjects were classified as having normal diastolic function (E/e′< 8), moderate diastolic dysfunction (8 < E/e′ < 12), or severe diastolic dysfunction (E/e′ ≥ 12) consistent with recommendations of the American Society of Echocardiography.^11^ Intra-observer variation (measured by blinded re-analysis of a random sample of 20% of recorded video files) was 5% for EF% and E/e′.

#### Myocardial fibrosis

Calibrated integrated backscatter (cIB) is a reproducible, noninvasive measure of ultrasonic tissue reflectivity and a validated marker of myocardial fibrosis.^13–15^ Briefly, a sampling cursor with a fixed region of interest was placed on the pericardium to record integrated pericardial backscatter (perIB [-dB]), which indexed reference fibrosis. For quantification of myocardial tissue reflectance, the sampling cursor was placed in the mid-myocardium of the anterior septum (sepIB) and posterior wall (postIB) of the LV and the measures recorded. The position of the sample volume was monitored and adjusted per frame to maintain the sample volume within the same region during the whole cardiac cycle. Next, cIB was calculated by subtracting refractive intensities, eg, [cIB_post_ = postIB – perIB] and [cIB_sept_= sepIB – perIB], and averaged per patient to obtain avcIB as a global indicator of myocardial fibrosis. Given the mathematic relationship to sepIB, values of avcIB closer to zero indicate a greater degree of fibrosis. Patients were classified as normal (avcIB < −15 dB) or presenting with myocardial fibrosis (avcIB > −15 dB).^13^

#### Exercise capacity

Symptoms of heart failure during everyday activities were assessed according to the New York Heart Association (NYHA) classification, based on specific questions pertaining to breathlessness during everyday activities and difficulties climbing stairs.^16^

Exercise testing was performed as described previously.^17^ Peak oxygen consumption (VO_2peak_) was measured using the modified Bruce treadmill protocol. After 15 minutes of rest and measurement of baseline heart rate and oxygen consumption, breath-by-breath flow and volume of inspired and expired gases were continuously monitored using a Medgraphics CardiO2 combinedVO_2_/ECG exercise system (St. Paul, MN) during progressively increasing treadmill speed and elevation angle. Subjects were constantly encouraged to complete 3-minute stages, and the test was terminated once peak volitional effort and peak heart rate were recorded. VO_2peak_ was normalized to body weight (ml/min/kg) for interindividual comparison.

#### Cytokines, catecholamines, and cortisol

Abundance of the following cytokines were determined as described elsewhere:^18^ IL-1β, IL-2, IL-4, IL-5, IL-6, IL-7, IL-8, IL-10, IL-12, IL-13, IL-17, TNFα, interferon γ, granulocyte-monocyte-colony stimulating factor (GM-CSF), monocyte chemoattractant protein-1, and macrophage inflammatory protein-1β. Concentrations of catecholamines (norepinephrine, epinephrine, dopamine) in urine, and cortisol in serum and urine per 24 hours were measured and recorded similarly. For statistical modelling, the maximum and mean levels of cytokines, catecholamines, and cortisol were calculated for the period of acute hospitalization and for the period between discharge and study date.

### Statistical analysis

Prior to enrollment, a power analysis was carried out to determine the number of subjects needed to demonstrate differences in systolic function from healthy controls. Based on a normal EF% of 60±20%, a hypothesized effect size of 15%, a type-I error rate (α) of 0.05, and power (1-β) of 0.8, it was determined that 39 burn subjects would need to be enrolled to detect a statistically significant differences in EF%. All analyses were carried out with R 3.3 for Windows (Vienna, Austria) or Graphpad Prism 7.00 for Windows (La Jolla, CA). Student’s t-test and one-way ANOVA were used to compare continuous outcomes. Standard univariate and multivariate least-squares regression models were fit to continuous responses. As necessary, predictors and responses were transformed to allow for better fitting of the model assumptions. For categorical outcomes, logistic regression models were fit; inference was based on comparisons of deviances among hierarchically fit models. Multi-variable logistic regression models were fit and assessed using standard generalized linear model functions in R. All data are reported as mean ± SD unless otherwise noted. For all analyses, statistical significance was reported with p < 0.05.

### Data sharing

R code and data of univariate and multivariate analyses can be accessed online through the Medeley network: http://dx.doi.org/10.17632/xrh3pd9by7.1

### Role of the funding source

None of the study sponsors had any role in the study design; in the collection, analysis, or interpretation of data; in the writing of the report; or in the decision to submit the paper for publication. The following authors had access to the raw data: GH, RPC, VNC, PW, AMQ, KJ, LKB, NRM, CCF, OES, DNH, MPK. The corresponding author had full access to all data in the study and had final responsibility for the decision to submit for publication.

## Results

As shown in table 1, the 40 burn subjects enrolled in this study were injured at 8 ± 5 years of age, had 59 ± 19% TBSA burns, and were examined for this study at 12 ± 4 years postburn. The burn subjects and the healthy control group had a comparable sex distribution and age at the time of the study. The ethnicity distribution of the study group was different from healthy controls, with the majority of subjects being Hispanic. There was no history of congenital cardiac disease in any of the study subjects, personal and family medical history were noncontributory regarding cardiovascular disease. There was no difference in BMI between the groups.

### Primary endpoints

**Figure 1:**
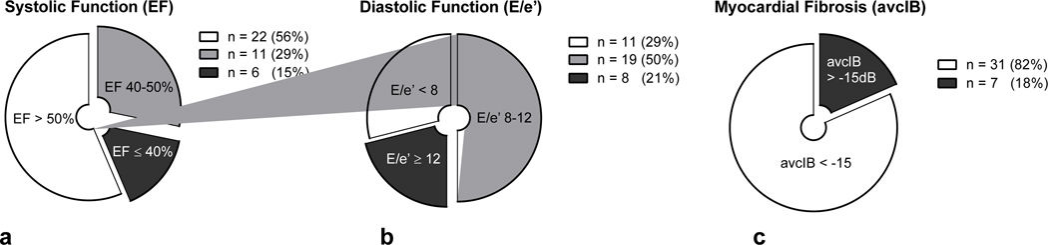
Distribution of long-term systolic function, diastolic function, and myocardial fibrosis in pediatric burn survivors EF = ejection fraction. E/e′ = ratio of E wave to e′—preload-independent index of LV compliance. avcIB = average calibrated integrated backscatter of myocardial septum and posterior LV wall.

**Figure 2:**
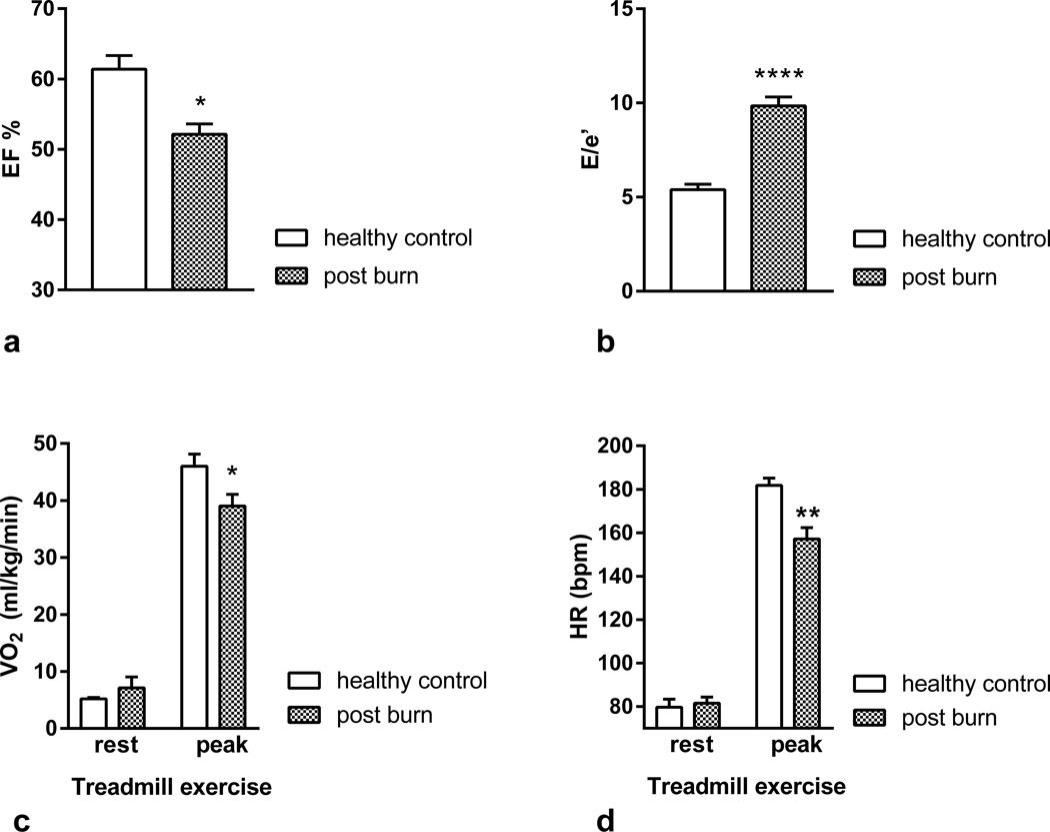
Long-term echocardiographic and functional data in pediatric burn survivors a: Ejection fraction (EF%). Burn: 52 ± 9.1; Control: 61 ± 6.1; *p < 0.01. b: Diastolic function (E/e′). Burn: 9.8 ± 2.9; Control: 5.4 ± 0.9; ****p < 0.0001. c: Oxygen consumption (VO_2_) adjusted to body weight at rest and under peak treadmill exercise. Peak: Burn, 37.9 ± 12 ml/min/kg; Control, 46 ± 8.32 ml/min/kg; *p < 0.05. d: Heart rate (bpm) at rest and under peak treadmill exercise. Peak: Burn, 161 ± 26; Control, 182 ± 13; **p < 0.01.

Echocardiographic findings concerning systolic and diastolic function, as well as myocardial fibrosis are summarized in table 2 and figure 1. EF% was lower in burn subjects (52 ± 9.1%) than in healthy controls (61 ± 6.1%, p=0.004; figure 2a). A substantial percentage of burn subjects presented with EF% below 50% (n=11, (28%)) and 40% (n=6, (15%)). E/e′ was impaired in burn subjects (9.8 ± 2.9) compared to healthy controls (5.4 ± 0.9, p < 0.0001; figure 2b). Moderate diastolic dysfunction (E/e′ = 8-12) was noted in 50% (n=19) of burn subjects and severe dysfunction (E/e′ ≥ 12) in 21% (n=8).

The avcIB of the septal and posterior wall was -22 ± 6 dB, and 18% of burn subjects were classified as presenting with myocardial fibrosis (avcIB > −15 dB). Significant correlations were detected between diastolic dysfunction and fibrosis (ie, E/e′ and avcIB; r = 0.448; p = 0.005) and systolic dysfunction and fibrosis (EF% and avcIB; r = −0.3458; p = 0.033). No significant correlation was found between systolic and diastolic dysfunction (EF% and E/e′; r = −0.2934; p = 0.07).

### Exercise capacity

Twenty-six burn subjects (65%) were classified as NYHA I, 13 (33%) as NYHA II, 1 (3%) as NYHA III and none as NYHA IV. No differences in resting VO_2_ or heart rate were detected between the groups (table 3, figures 2c and d). Exercise testing revealed that burn subjects had lower absolute VO_2_ peak (burn: 2469 ± 901ml/min vs. control: 3150 ± 662ml/min; p = 0.02) and weight-adjusted VO_2_ peak (burn: 38 ± 12 ml/min/kg vs. control: 46 ± 8 ml/min/kg; p = 0.03). They also had a lower absolute heart rate increase (burn: 86 ± 32 bpm vs. control: 102 ± 15 bpm; p = 0.018), relative heart rate increase (burn: + 99.8 ± 58.6% vs. control: 133 ± 36.2%; p = 0.047), and peak heart rate (burn: 161 ± 26 bpm vs. control: 182 ± 13 bpm; p = 0.006).

### Predictors of primary endpoints

1. Clinical variables: Length of hospitalization (effect: 0.0805; p=0.048), ventilation days (log; effect: 2.09, p = 0.017), TBSA burned (effect: 0.175, p = 0.003), and Baux score (effect: 0.127, p = 0.006) had significant least squares effects on long-term myocardial fibrosis; Baux score had a significant logistic effect on the categorical outcome of fibrosis (avcIB < −15 dB; OR: 1.1, p = 0.025) (supplemental table 1). Administration of any inotrope and dobutamine in particular were associated with increased probability of severe systolic dysfunction (EF < 40%; OR: 10.83, p = 0.012 and OR: 7.2, p = 0.037). Acute administration of dobutamine predicted E/e′ > 8 (OR: 11, p = 0.002). No significant least squares or logistic effects were detected for sex, burn etiology, time postburn, delay of admission, number of acute operations, sepsis, or type of research medication on any of the echocardiographic endpoints.
2. Cytokine concentrations: Linear regression of cytokine concentrations measured during intensive care hospitalization showed significant association between maximal levels of IL-1β (effect: −18.7; p = 0.038), TNFα (effect: −0.025, p = 0.045), and G-CSF (effect: −0.003, p = 0.033) and EF% as well as mean levels of TNFα (log; effect: −3.53, p = 0.0113) and EF% (supplemental table 2). There was an effect of acute mean IL-8 on E/e′ (log; effect: 1.45, p = 0.044). Least squares regression of cytokine concentrations measured between discharge from acute hospitalization and echocardiographic evaluation showed an effect of mean serum cortisol levels (log; effect: −6.49, p < 0.05), maximum IL-2 (effect: 0.049, p = 0.015), mean IL-2 (effect: 0.201, p = 0.0013), maximum GM-CSF (log, effect: 2.77, p = 0.005), and mean GM-CSF (log, effect: 3.6, p = 0.005) on avcIB (supplemental table 3).
  Logistic regression of acute cytokine concentrations showed that maximum IL-5 (log; OR: 4.91, p = 0.015) and mean IL-5 (log; OR: 6.13, p = 0.031) were significant predictions of EF% < 40%. Maximum IL-8 (log; OR: 4.29, p = 0.032) and mean IL-8 (log; OR: 11.8, p = 0.013) during acute hospitalization predicted an abnormal E/e′ > 8. Logistic regression yielded no other significant results for any of the other analyzed parameters, time points, and outcomes.
3. Multivariate modelling: Multi-variable logistic regression models (table 3) showed significant predictive power of acute maximum [IL-1β, IL-6, IL-8, TNFα] for the long-term events EF% < 50% (p = 0.023), E/e′ > 8 (p < 0.0016) and acute mean [IL-1β, IL-6, IL-8, TNFα] for the long term events EF% < 50% (p = 0.0063), E/e′ > 8 (p < 0.0001). Acute maximum [IL-1β, IL-6, IL-8, TNFα+ predicted EF% < 40% (p = 0.0299) and avcIB > −15dB (p = 0.0267). Acute mean [epinephrine, cortisol] predicted EF < 50% (p = 0.0499) and EF < 40% (p = 0.0293).

## Discussion

This study provides evidence for fibrosis and long-term cardiac dysfunction in young adults who survive severe thermal trauma as a child. Here we have established long-term associations between injury severity and systemic inflammatory stress on one hand and specific indicators of myocardial fibrosis and systolic and diastolic dysfunction on the other.

Almost half (43%) of the long-term burn survivors examined in this study showed signs of systolic dysfunction with an EF% below 50%, and the average EF% was substantially lower than that in healthy controls. Our data suggest that acute depression of contractility, as described in various reports on pediatric and adult burn injury and other critical illness, may persist longer than originally anticipated.^19^ may not be fully reversible, as suggested in reports emphasizing temporary dysfunction instead of structural impairment.^20^ Systolic dysfunction in young adults has important implications, as large prospective trials identified it as a powerful predictor of heart failure.^10^

LV diastolic dysfunction characteristically results in increased LV filling pressure.^11^ A considerable proportion of burn subjects displayed signs of moderate (50%) or severe (21%) LV diastolic dysfunction, and average diastolic function was significantly worse than in healthy controls. Secondary findings of decreased E/A ratio and increased pulmonary capillary wedge pressure support the presence of increased LV filling pressure. LV diastolic dysfunction develops early in most cardiac diseases, has a high prognostic value, and is an important indicator for LV heart failure in the absence of systolic abnormalities.^21^ In young adults it has been identified as an independent predictor for the early development of heart failure, reduced exercise capacity, and increased mortality.^22,23^

We found that 20% of the study population showed evidence of myocardial fibrosis, when using a conservative cut-off for average LV and septal integrated backscatter of -15dB.^14,24,25^ Increased LV filling pressure, systolic and diastolic dysfunction and myocardial fibrosis interact with and promote one another: Deregulated interstitial collagen I synthesis and deposition occur as a result of increased ventricular pressure, induce subsequent diastolic dysfunction through ventricular stiffening, which also worsens LV systolic function.^26,27^ The significant correlations between avcIB, EF% and E/e′, underscore this interdependence. The presence of diastolic dysfunction and myocardial fibrosis is associated with poor prognosis, a greater risk of death and a further decline in cardiac function.^28^

Echocardiographic findings in our study population were associated with impaired exercise tolerance – a hallmark of diastolic dysfunction.^29^ At rest, few burn survivors showed any of symptoms of heart failure while peak oxygen consumption during exercise was significantly reduced. Resting diastolic function has been established as the strongest echocardiographic correlate of exercise tolerance, while systolic function plays a minor role.^29^ Peak heart rate, as well as the absolute and relative increase in heart rate were reduced in burn patients. While the exact mechanism remains unclear, chronotropic failure (defined as the inability to reach target heart rates during strenuous exercise) has been shown to reliably predict mortality and incident cardiac disease in adults with clinically asymptomatic heart failure.^30^ We have previously proven prolonged elevation of resting heart rates and systemic catecholamines in pediatric burn survivors for more than two years postburn; a chronic down-regulation of beta-adrenergic receptors may contribute to the inability to increase heart rates and meet oxygen demand during exercise^3^.

Whether our long-term findings are associated with critical illness in general or burn trauma in particular is unclear. Echocardiographic markers of fibrosis were associated with general clinical measures of critical illness severity, such as length of hospitalization, ventilation days, and TBSA burned. The administration of inotrope medication in general and dobutamine in particular significantly increased the probability of long-term severe systolic and mild diastolic dysfunction, suggesting that the severity of initially sustained cardiac strain may play an important role in long term dysfunction.^19^ In line with studies, that suggest systemic stress and inflammation as inductors of cardiovascular sequelae^31^, we found significant associations between acutely elevated pro-inflammatory cytokines and measures of systolic and diastolic dysfunction. Systemic inflammation has been linked to LV hypertrophy, collagen I deposition, and ventricular stiffening.^31^ In line with our multivariate models, a number of reports have identified TNFα, IL-1β, and IL-6 as main drivers of acute cardiac depression and long-term structural remodeling.^1,7^

The implications of our findings are difficult to gauge at present. Data by Duke et al. suggest that adult and pediatric burn survivors are more prone to sequelae of cardiovascular disease later in life.^8,9^ However, no prospective studies have followed a cohort of survivors to assess the progression of morphologic and functional changes. Based on data showing systolic and diastolic dysfunction as well as myocardial fibrosis to be independent risk factors for poor cardiovascular and overall health, it is reasonable to assume that, in children surviving burn injury, cardiovascular disease burden may increase disproportionally later in life.

This study has limitations that bear consideration. The observational and cross-sectional nature of this study prevents conclusions of a prospective, longitudinal trial. This is emphasized by the fact, that no consistent echocardiographic baseline data of cardiac function during acute hospitalization was available for analysis. Therefore, no inferences can be made regarding the exact trajectory and possible persistence of cardiac dysfunction. As a consequence of this study, structured and prospective assessments of the presented and additional endpoints, spanning time points from the acute phase to long term recovery have been implemented at our institution to elucidate the trajectory of cardiac dysfunction and remodeling more comprehensively over the next decades. While the repeated hospitalization for reconstructive procedures of our study subjects enabled this study in the first place, the implicated systemic strain itself may have contributed negatively to the observed results. Systolic and diastolic function measurements could not be obtained in two subjects due to hypertrophic scarring of the chest. The group of healthy volunteers was ethnically more diverse than the group of burn patients; except BMI, no objective measures of cardiac risk factors independent of burn injury were assessed; mental status^32^ or quality of life post burn were not evaluated as possible contributors to cardiac dysfunction. Data on specific sports activity was not collected. Future studies will need to include this variable, as exercise may have positive effects on the progression of cardiac dysfunction. Also, due to the novelty of the observed data, our univariate analyses are broad and bound to the type and timing of measurements made in the past. This study was underpowered to confirm the established linear relationship between diastolic dysfunction, fibrosis and exercise intolerance.

Clearly, well-powered prospective trials with systematic assessments from the acute phase through long-term time points are needed to elucidate all complex mechanisms at play. Such research, also in other fields of pediatric and adult critical care, will ultimately assess the generalizability of our findings.

## Conclusions

Long-term survivors of severe pediatric burn injury show signs of systolic and diastolic dysfunction as well as evidence of structural cardiac remodeling, and resultant exercise intolerance. Surrogate parameters of inflammation and severity of critical illness are associated with extent of cardiac dysfunction in young adults. The implications of these findings for future cardiovascular disease burden in these patients are unclear at present.

## Supplementary Material

supplement

## Figures and Tables

**Table 1:** Study subject characteristics

Characteristic	Postburn (N = 40)	Healthy Control (N = 25)	p
Age at study (years)	19±5	21±3	ns
Age at burn (years)	8±5	–	–
Years post burn	12±4	–	–
Sex (male/female)	23/17	11/14	ns
Ethnicity			
Hispanic – Latino	36 (90)	5	< 0.0001
White American	4 (10)	10	
African American/Asian	0 (0)	8	
BMI (kg/m2)	24±4	23±5	ns
TBSA burned (%)	59±19	–	–
Baux score	74±25	–	–
Cause of burn			
Flame	30 (75)	–	–
Scald	5 (12.5)	–	–
Electrical injury	5 (12.5)	–	–
DA (days)	6±8	–	–
LOH (days)	39±29	–	–
Inhalation injury	16 (40)	–	–
Days on mechanical ventilation	12±23	–	–
Acute operations	6±5	–	–
Sepsis	9 (23)	–	–
Inotrope medication administered	14 (35)		
Dobutamine	10 (25)	–	–
Epinephrine	2 (5)	–	–
Dopamine	1 (2.5)	–	–
Milrinone	1 (2.5)	–	–

Data reported as mean ± SD unless or n (%) unless otherwise noted.

BMI = Body mass index. Baux score = patient age + TBSA burned + 17 (if inhalation injury present). TBSA = total body surface area. DA = delay of admission (days from burn to admission). LOH = length of acute hospitalization (days).

**Table 2:** Echocardiographic results

Measurement	Postburn	Healthy Control	p
Systolic function			
EF, %	52 ± 9.1	61 ± 6.1	0.004
EF < 50%	11 (28)	0 (0)	
EF < 40%	6 (15)	0 (0)	
Diastolic function			
E/e′	9.8 ± 2.9	5.4 ± 0.9	< 0.0001
E/e′ 8–12	19 (50)	0 (0)	
E/e′ > 12	8 (21)	0 (0)	
E/A	1.8 ± 0.5	–	
E/A ≥ 2	13 (34)	–	
TR jet (m/s)	1.7 ± 1.3	–	
Integrated backscatter			
postcIB	−21 ± 4	–	
sepcIB	−24 ± 8	–	
avcIB (dB)	−22 ± 6	–	
avcIB > −15 dB	7 (18)	–	
PCWP (mmHg)	13.8 ± 4.1	8.6 ± 1.46	0.0003
PCWP > 15	15 (39)	0 (0)	

Correlations	r	R2	p

E/e′ – avcIB	0.4481	0.2	0.005
EF – avcIB	−0.3458	0.12	0.033
EF – E/e′	−0.2934	0.086	0.07

Data reported as mean ± SD or n (%).

EF = ejection fraction. E/e′ = ratio of E-wave to e′. E/A = ratio of early and late LV diastolic filling velocity. TR jet = tricuspid regurgitation velocity. postcIB = calibrated integrated backscatter of the posterior LV wall. sepcIB = calibrated integrated backscatter of septal LV wall. avcIB = average calibrated integrated backscatter of septal and posterior LV wall. PCWP = pulmonary capillary wedge pressure (calculated via the Nagueh-formula: PCWP = 1.24 * (E/e′) + 1.9; Nagueh et al. 1997). E/A, TR jet, and integrated backscatter were not assessed in the control group.

**Table 3:** Multivariable regression models of proinflammatory cytokines, catecholamines and cortisol for long-term outcomes

	Long-term event							

	EF < 50%		EF < 40%		E/e′ > 8		avcIB	

	Estimate	p	Estimate	p	Estimate	p	Estimate	p
Model [IL-1b, TNFα, IL-6, IL-8]								
Acute, maximum concentration								
Intercept	−0.98	0.212			−2.12	0.223	−1.97	0.0502
IL-1P	0.0183	0.556			0.00266	0.651	−0.0897	0.204
IL-6	0.00144	0.193			−0.000452	0.554	0.00141	0.0407
IL-8	−0.000487	0.299			0.0168	0.0542	#	#
TNFα	0.0156	0.392			−0.0169	0.408	−0.00367	0.221

R^2^	0.438	**0.0232**			0.596	**0.00159**	0.427	**0.0267**

Model [IL-1b, TNFα, IL-6, IL-8]								
Acute, mean concentration								
Intercept	−2.08	0.0506	−2.09	0.0384	−3.28	0.0867		
IL-1β	0.055	0.544	−0.0467	0.116	−0.00811	0.824		
IL-6	−0.000432	0.114	−0.00138	0.502	0.000419	0.867		
IL-8	0.00525	0.754	#	#	0.0381	0.0196		
TNFα	0.121	0.122	0.167	0.0311	−0.024	0.728		

R^2^	0.494	**0.0063**	0.356	**0.0299**	0.633	**< 0.0001**		

Model [epinephrine, cortisol]								
Acute, mean concentration								
Intercept	0.257	0.805	−0.536	0.67				
Epinephrine	0.00814	0.401	0.0104	0.448				
Cortisol	−0.00511	0.211	−0.00547	0.327				

R^2^	0.683	**0.0499**	0.631	**0.0293**				

# = Modeled without IL-8. Epinephrine and cortisol concentrations per 24h in urine, averaged over duration of acute hospitalization.

## List of Abbreviations

A wave: mitral inflow velocity of active filling via atrial contraction
avcIB: average calibrated integrated backscatter
cIB: calibrated integrated backscatter
DA: delay of admission
E wave: mitral inflow velocity
e′: relaxation velocity of mitral annulus
EDV: end-diastolic volume
E/e′: ratio of E wave to e′—index of LV compliance
EF%: ejection fraction
ESV: end-systolic volume
GM-CSF: granulocyte-monocyte-colony stimulating factor
IL: interleukin
LOH: length of acute hospitalization
LV: left ventricle
PCWP: pulmonary capillary wedge pressure
perIB: pericardial integrated backscatter
postIB: posterior LV wall integrated backscatter
sepIB: septal wall integrated backscatter
TNFα: tumor necrosis factor alpha
V_O2peak_: peak oxygen consumption
