# Supplementary material for: Long-term effect of critical illness after severe paediatric burn injury on cardiac function in adolescent survivors: an observational study
Source: Lancet Child Adolesc Health. Author manuscript; Available in PMC 2018 Dec 1. (PMC5865217; doi:10.1016/S2352-4642(17)30122-0)
Supplement: supplement [file NIHMS917690-supplement.pdf]

Parameters of acute stay		Linear regression					
		EF		E/e'		avcIB	
		means	p value	means	p value	means	p value
Sex			0·209		0·44		0·69
	M	50·1		10·1		22·7	
	F	53·9		9·38		21·7	
Cause of burn			0·937		0·0621		0·314
	Flame	51·6		10·5		21·3	
	Scald	51		8·18		26·4	
	Electrical	53		7·74		23·6	
Acute study drug			0·933		0·275		0·317
	Placebo	51		10·8		23·3	
	Oxandrolone	55·2		9·8		23·5	
	Propanolol	50·9		10		22·3	
	Other	53·2		6·9		19	
		slopes	p value	slopes	p value	slopes	p value
Age at burn		-0·0855	0·764	-0·0886	0·34	-0·0335	0·883
Length of hospitalization (days)		-0·0705	0·127	0·00967	0·526	0·0805	**0·048**
Time postburn (years)		0·0858	0·803	0·0248	0·825	0·383	0·179
Burn to admit (days)		0·134	0·502	-0·0324	0·618	0·0159	0·926
	log	2·24	0·267	-0·583	0·376	-2·3	0·15
Mechanical ventilation days		-0·0657	0·308	0·0206	0·329	0·0867	0·0995
	log	-1·65	0·141	0·597	0·108	2·09	**0·0171**
Number of operations		-0·437	0·181	0·0452	0·674	0·613	0·0685
Percent TBSA burned		-0·12	0·145	-0·00549	0·841	0·175	**0·0033**
Percent TBSA full-thickness burned		-0·0686	0·274	0·000757	0·971	0·0874	0·0589
Baux-score		-0·0886	0·15	-0·00515	0·802	0·127	**0·0056**

Logistic regression

		EF < 50%		EF < 40%		E/e' > 8		E/e' ≥ 12		avcIB > -15dB	
		prob	p value	prob	p value	prob	p value	prob	p value	prob	p value
Sex			0·342		0·37		0·728		1		1
	M	0·478		0·217		0·652		0·217		0·136	
	F	0·312		0·0625		0·733		0·2		0·125	
Cause of burn			0·862		1		0·00697		1		0·592
	Flame	0·379		0·172		0·821		0·214		0·179	
	Scald	0·6		0·2		0·4		0·2		0	
	Electrical	0·4		0		0·2		0·2		0	
Acute study drug			0·833		0·934		0·151		1		0·226
	Placebo	0·5		0·167		0·833		0·25		0·0909	
	Oxandrolone	0·25		0		0·75		0·25		0	
	Propanolol	0·389		0·222		0·706		0·235		0·111	
	Other	0·4		0		0·2		0		0·4	
		OR	p value	OR	p value	OR	p value	OR	p value	OR	p value
Age at burn		1·13	0·15	1·14	0·134	1·11	0·308	0·988	0·895	1·17	0·242
Length of hospitalization (days)		1·03	0·0666	1·02	0·174	1·02	0·174	0·996	0·788	1·01	0·289
Time postburn (years)		0·906	0·205	0·93	0·483	0·998	0·976	1·05	0·629	1·06	0·612
Burn to admit (days)		0·989	0·811	0·816	0·309	0·954	0·293	0·984	0·788	1·02	0·799
	log	0·662	0·381	0·329	0·203	0·475	0·121	0·658	0·484	0·712	0·632
Mechanical ventilation days		1·03	0·142	1·01	0·648	1·17	0·0807	0·989	0·623	1·02	0·348
	log	1·46	0·15	1·53	0·214	2·45	**0·0152**	1·12	0·708	1·5	0·271
Number of operations		1·13	0·15	1·14	0·134	1·11	0·308	0·988	0·895	1·17	0·242
Percent TBSA burned		1·02	0·299	1·04	0·113	1·02	0·26	0·986	0·538	1·07	**0·0376**
Percent TBSA full-thickness burned		1·01	0·48	1·05	0·0551	1·01	0·408	0·998	0·926	1·03	0·14
Baux-score		1·01	0·389	1·03	0·0945	1·02	0·203	0·989	0·523	1·1	**0·0252**
Sepsis		0·64	0·7023	0·595	0·999	3·5	0·395	0·172	0·35	0·686	0·999
Any inotrope administered		2·71	0·285	10·83	**0·012**	5·88	0·124	1·286	0·999	1·438	0·6527
Dobutamine administered		1·635	0·706	7·2	**0·037**	11	**0·002**	1·095	0·999	2.083	0.591

EF = ejection fraction. E/e’ = ratio of E-wave to e’. avcIB = average calibrated integrated backscatter of septal and posterior LV wall. TBSA = total body surface area. Baux score = patient age + TBSA burned + 17 (if inhalation injury present). Prob: Probability. OR: Odds ratio.

Supplemental Table 1: **Linear and logistic regression of parameters of acute hospitalization**

Parameters of acute stay			Linear regression					
			EF		E/e'		avcIB	
			slope	p value	slope	p value	slope	p value
Cortisol (µg/24h, Urine)		max	0·0114	0·103	-0·00148	0·453	0·0016	0·739
	log		3·64	0·251	-0·0891	0·92	2·03	0·336
		mean	0·015	0·221	-0·00211	0·537	0·00456	0·579
	log		2·44	0·493	-0·237	0·81	2·44	0·294
Cortisol (pg/ml, Serum)		max	0·0705	0·501	0·021	0·737	0·0544	0·694
	log		1·79	0·651	0·397	0·867	3·12	0·549
		mean	0·112	0·569	0·0278	0·813	0·0458	0·87
	log		1·96	0·689	0·0443	0·988	1·82	0·786
NEPI (µg/24h, Urine)		max	0·013	0·366	-0·000995	0·84	-0·00968	0·317
	log		0·366	0·866	-0·198	0·789	-0·564	0·698
		mean	0·0163	0·48	-0·00245	0·753	-0·0104	0·503
	log		-0·078	0·972	-0·324	0·671	-0·266	0·861
EPI (µg/24h, Urine)		max	-0·00534	0·184	0·000464	0·736	0·000322	0·907
	log		-1·01	0·583	-0·297	0·63	-1·09	0·377
		mean	-0·0157	0·121	0·00142	0·684	0·000128	0·985
	log		-1·41	0·476	-0·405	0·544	-1·17	0·381
DOPA (µg/24h, Urine)		max	0·0181	0·111	-0·0036	0·358	-0·00597	0·446
	log		3·45	0·238	-0·697	0·488	-0·99	0·62
		mean	0·0216	0·208	-0·00482	0·409	-0·00834	0·478
	log		2·69	0·399	-0·523	0·629	-1·4	0·514
IL-1β (pg/ml)		max	-0·0074	0·104	-0·0019	0·19	-0·00239	0·449
	log		-1·87	**0·0383**	-0·18	0·545	-0·556	0·382
		mean	-0·0383	0·132	-0·0104	0·197	-0·0121	0·49
	log		-2·34	0·0623	-0·38	0·352	-0·899	0·303
IL-2 (pg/ml)		max	-0·00204	0·95	-0·0168	0·0949	0·0296	0·176
	log		-1·45	0·288	-0·386	0·385	1·68	0·0666
		mean	-0·0174	0·949	-0·138	0·1	0·189	0·303

	log		-1·29	0·547	-0·714	0·298	1·54	0·292
IL-4 (pg/ml)		max	0·0105	0·145	0·00293	0·202	0·00187	0·708
	log		0·573	0·628	-0·0284	0·941	0·415	0·609
		mean	0·0626	0·146	0·0177	0·196	0·0106	0·722
	log		1·65	0·341	-0·0338	0·952	0·484	0·684
IL-5 (pg/ml)		max	-0·459	0·0763	-0·076	0·363	-0·108	0·549
	log		-2·56	0·171	-0·22	0·716	0·0564	0·965
		mean	-0·591	0·507	-0·393	0·156	-0·364	0·548
	log		-3·54	0·239	-0·944	0·323	-0·216	0·917
IL-6 (pg/ml)		max	-0·00067	0·183	-0·000122	0·447	-0·000070	0·839
	log		-0·619	0·641	0·173	0·683	1·4	0·11
		mean	-0·00281	0·253	-0·000494	0·527	-0·00027	0·873
	log		-0·744	0·668	0·212	0·702	1·67	0·146
IL-7 (pg/ml)		max	0·0127	0·816	-0·0325	0·0517	-0·0271	0·468
	log		-0·92	0·557	-0·52	0·29	0·228	0·85
		mean	0·0661	0·59	-0·0526	0·168	-0·051	0·544
	log		-0·556	0·762	-0·78	0·171	-0·142	0·918
IL-8 (pg/ml)		max	-0·00389	0·111	0·000919	0·238	0·0018	0·29
	log		-3·07	0·104	1·07	0·0742	2·21	0·107
		mean	-0·0141	0·162	0·0041	0·201	0·0047	0·506
	log		-3·37	0·147	1·45	**0·044**	1·9	0·266
IL-10 (pg/ml)		max	-0·00584	0·334	0·000335	0·862	-0·000705	0·865
	log		-1·83	0·09	0·0211	0·952	0·316	0·681
		mean	-0·0289	0·333	0·00133	0·889	-0·00405	0·843
	log		-2·11	0·16	0·0262	0·957	0·0719	0·945
IL-12 (pg/ml)		max	-0·00413	0·843	-0·0108	0·0918	0·014	0·32
	log		-1·64	0·21	-0·545	0·201	0·493	0·591
		mean	-0·0313	0·823	-0·0834	0·0507	0·0791	0·402
	log		-2·24	0·268	-1·14	0·0747	0·308	0·826
IL-13 (pg/ml)		max	0·0671	0·675	-0·0732	0·141	-0·122	0·257
	log		0·597	0·74	-0·899	0·105	-1·03	0·396
		mean	0·474	0·126	-0·0876	0·379	-0·214	0·316

	log		2·47	0·332	-0·99	0·217	-2·39	0·162
IL-17 (pg/ml)		max	-0·00584	0·334	0·000335	0·862	-0·000705	0·865
	log		-1·83	0·09	0·0211	0·952	0·316	0·681
		mean	-0·0289	0·333	0·00133	0·889	-0·00405	0·843
	log		-2·11	0·16	0·0262	0·957	0·0719	0·945
GM-CSF (pg/ml)		max	-0·0154	0·7	0·00264	0·837	0·0136	0·615
	log		-0·719	0·581	-0·0381	0·93	0·968	0·299
		mean	-0·029	0·847	0·0206	0·669	0·0164	0·874
	log		-0·998	0·536	0·0175	0·974	0·624	0·587
INFγ (pg/ml)		max	-0·0146	**0·0365**	-0·0034	0·133	-0·00306	0·535
	log		-1·44	0·16	-0·169	0·608	0·321	0·66
		mean	-0·0569	**0·0499**	-0·0112	0·236	-0·012	0·555
	log		-2·02	0·114	-0·233	0·581	0·0109	0·991
TNFα (pg/ml)		max	-0·0249	**0·0454**	-0·00589	0·142	-0·00706	0·418
	log		-2·1	0·0553	-0·468	0·187	-0·49	0·535
		mean	-0·0672	0·0705	-0·0147	0·218	-0·0176	0·497
	log		-3·53	**0·0113**	-0·6	0·199	-0·842	0·405
G-CSF (pg/ml)		max	-0·00299	**0·0461**	-0·0000668	0·892	0·000427	0·687
	log		-2·25	0·0907	0·000433	0·999	1·05	0·277
		mean	-0·0164	**0·0331**	-0·00118	0·645	0·00138	0·802
	log		-2·65	0·0808	-0·0908	0·858	0·752	0·51
MCP-1 (pg/ml)		max	-0·00256	0·346	-0·000782	0·362	0·00113	0·548
	log		-2·18	0·27	-0·45	0·476	0·969	0·492
		mean	-0·0000154	0·998	-0·00173	0·341	0·000231	0·954
	log		-0·964	0·657	-0·516	0·45	0·295	0·852
MIP-1b (pg/ml)		max	-0·00772	0·572	-0·00226	0·6	-0·00221	0·817
	log		-1·32	0·537	-0·147	0·829	0·52	0·749
		mean	-0·011	0·712	-0·000719	0·939	-0·0145	0·487
	log		-1·32	0·605	-0·0394	0·961	-0·608	0·761

EF = ejection fraction. E/e’ = ratio of E-wave to e’. avcIB = average calibrated integrated backscatter of septal and posterior LV wall. NEPI = norepinephrine. EPI = epinephrine. DOPA = dopamine. OR = odds ratio.

***Supplemental table 2a:* Linear regression of biomarkers during acute hospitalization**

**Parameters of acute stay**		**Logistic regression**									
		EF < 50%		EF < 40%		E/e' > 8		E/e' ≥ 12		avcIB > -15	
		OR	p value	OR	p value	OR	p value	OR	p value	OR	p value
Cortisol (µg/24h, Urine)	max	0·999	0·46	0·998	0·438	0·999	0·501	0·998	0·419	1	0·358
	log	0·829	0·769	0·718	0·652	1·12	0·868	0·683	0·58	4·8	0·208
	mean	0·998	0·555	0·999	0·687	0·999	0·657	0·997	0·375	1	0·22
	log	0·94	0·93	0·875	0·866	1·08	0·914	0·591	0·495	8·1	0·185
Cortisol (pg/ml, Serum)	max	0·972	0·511	1·02	0·742	1·01	0·778	1	1	0·971	0·386
	log	0·36	0·482	3·21	0·589	1·04	0·972	0·688	0·721	0·495	0·534
	mean	0·939	0·433	1·03	0·753	1·01	0·811	0·975	0·635	0·919	0·242
	log	0·225	0·395	3·65	0·633	0·77	0·845	0·406	0·493	0·225	0·315
NEPI (µg/24h, Urine)	max	0·997	0·398	0·998	0·568	1	0·779	0·992	0·242	0·997	0·585
	log	1·01	0·985	1·22	0·692	0·838	0·723	0·56	0·244	0·806	0·72
	mean	0·995	0·386	0·996	0·544	1	0·97	0·991	0·252	0·998	0·825
	log	1·04	0·938	1·14	0·799	0·735	0·561	0·583	0·277	0·98	0·974
EPI (µg/24h, Urine)	max	1	0·392	1	0·267	1·01	0·466	0·999	0·601	0·984	0·466
	log	1·23	0·572	1·17	0·696	1·48	0·454	0·504	0·218	0·599	0·469
	mean	1·01	0·285	1	0·283	1·01	0·651	1	0·951	0·961	0·342
	log	1·36	0·448	1·13	0·784	1·33	0·586	0·551	0·286	0·537	0·444
DOPA (µg/24h, Urine)	max	0·998	0·489	1	0·859	0·997	0·221	0·996	0·246	0·997	0·475
	log	0·717	0·579	1·19	0·795	0·422	0·254	0·459	0·239	0·621	0·567
	mean	0·998	0·54	0·999	0·865	0·996	0·297	0·994	0·223	0·997	0·57
	log	0·791	0·714	1·15	0·847	0·572	0·46	0·48	0·291	0·539	0·496
IL-1β (pg/ml)	max	1	0·449	1	0·11	0·998	0·148	0·981	0·561	0·871	0·33
	log	1·31	0·172	1·63	**0·0373**	0·851	0·393	0·861	0·527	0·549	0·239
	mean	1	0·424	1·01	0·174	0·982	0·277	0·983	0·619	0·533	0·377
	log	1·44	0·191	1·75	0·0651	0·763	0·302	0·794	0·508	0·243	0·237
IL-2 (pg/ml)	max	0·997	0·665	1	0·834	0·989	0·332	0·959	0·377	1·02	0·384
	log	1·11	0·689	1·5	0·215	0·888	0·68	0·794	0·501	1·96	0·103
	mean	0·988	0·83	1·01	0·921	0·919	0·242	0·941	0·566	1·14	0·127

	log		1·25	0·587	1·31	0·574	0·77	0·553	0·715	0·537	2·77	0·089
IL-4 (pg/ml)		max	0·988	0·516	0·997	0·746	1	0·654	1	0·378	1	0·897
	log		0·85	0·506	1·11	0·704	0·833	0·449	1·28	0·327	1·2	0·541
		mean	0·94	0·581	0·974	0·808	1·01	0·653	1·02	0·405	0·998	0·872
	log		0·76	0·49	0·906	0·83	0·773	0·459	1·45	0·307	1·35	0·432
IL-5 (pg/ml)		max	1·12	0·1	1·2	**0·0209**	0·963	0·471	0·949	0·491	0·958	0·656
	log		1·7	0·171	4·91	**0·015**	0·848	0·673	1·01	0·973	1·04	0·941
		mean	1·21	0·306	1·39	0·0918	0·889	0·503	0·793	0·427	0·84	0·617
	log		2·21	0·201	6·13	**0·0309**	0·758	0·652	0·764	0·706	0·821	0·824
IL-6 (pg/ml)		max	1	0·408	1	0·317	1	0·319	1	0·735	1	0·893
	log		0·939	0·806	1·59	0·216	0·852	0·574	1·33	0·37	1·31	0·493
		mean	1	0·421	1	0·192	0·999	0·327	1	0·784	1	0·987
	log		0·964	0·912	1·62	0·286	0·825	0·6	1·48	0·347	1·56	0·393
IL-7 (pg/ml)		max	1	0·978	1·01	0·292	0·992	0·442	0·972	0·158	0·999	0·927
	log		1·13	0·694	1·71	0·286	0·889	0·725	0·833	0·585	0·931	0·883
		mean	1	0·988	1·02	0·402	0·999	0·957	0·944	0·209	0·996	0·905
	log		1·17	0·666	1·55	0·391	0·891	0·764	0·694	0·36	0·952	0·93
IL-8 (pg/ml)		max	1	0·369	1	0·133	1	0·0846	1	0·58	0·999	0·53
	log		1·8	0·15	2·71	0·0582	4·29	**0·0321**	1·26	0·59	0·874	0·821
		mean	1	0·21	1	0·519	1·03	**0·0186**	1	0·915	0·999	0·727
	log		2·63	0·0759	1·97	0·254	11·8	**0·013**	1·38	0·55	0·916	0·903
IL-10 (pg/ml)		max	1·02	0·203	0·999	0·784	1	0·642	0·978	0·434	0·998	0·788
	log		1·31	0·243	1·5	0·135	1·06	0·817	0·773	0·369	1·14	0·671
		mean	1·07	0·34	0·997	0·777	1·01	0·63	0·971	0·692	0·991	0·778
	log		1·63	0·178	1·24	0·514	1·12	0·737	0·767	0·536	1·17	0·683
IL-12 (pg/ml)		max	0·999	0·761	1	0·619	0·993	0·304	0·938	0·477	1·01	0·169
	log		1·18	0·525	1·75	0·0784	0·843	0·536	0·684	0·323	1·44	0·277
		mean	0·993	0·805	1·01	0·659	0·928	0·274	0·84	0·483	1·06	0·139
	log		1·35	0·462	1·76	0·203	0·688	0·372	0·497	0·313	1·83	0·207
IL-13 (pg/ml)		max	1	0·897	1·02	0·504	0·994	0·863	0·765	0·283	0·983	0·762
	log		1·04	0·901	1·36	0·441	0·762	0·451	0·403	0·158	1·04	0·945
		mean	0·952	0·558	0·928	0·671	1·03	0·749	0·637	0·302	0·836	0·67

	log		0·969	0·949	0·94	0·922	0·89	0·822	0·263	0·194	0·649	0·639
IL-17 (pg/ml)		max	1·02	0·203	0·999	0·784	1	0·642	0·978	0·434	0·998	0·788
	log		1·31	0·243	1·5	0·135	1·06	0·817	0·773	0·369	1·14	0·671
		mean	1·07	0·34	0·997	0·777	1·01	0·63	0·971	0·692	0·991	0·778
	log		1·63	0·178	1·24	0·514	1·12	0·737	0·767	0·536	1·17	0·683
GM-CSF (pg/ml)		max	1	0·645	1·01	0·235	1	0·866	1	0·886	0·997	0·818
	log		1·09	0·72	1·09	0·797	1·01	0·96	1·01	0·965	1·16	0·713
		mean	1·02	0·595	1·01	0·838	1·01	0·691	1·01	0·767	0·969	0·548
	log		1·29	0·43	0·961	0·918	1·19	0·624	0·958	0·91	1·04	0·933
INFγ (pg/ml)		max	1	0·232	1·01	0·0891	0·997	0·226	0·995	0·319	0·997	0·541
	log		1·17	0·445	1·65	0·118	0·882	0·572	1·02	0·936	0·817	0·5
		mean	1·02	0·19	1·01	0·235	0·99	0·287	0·99	0·546	0·964	0·32
	log		1·38	0·227	1·48	0·248	0·908	0·726	1·02	0·945	0·707	0·389
TNFα (pg/ml)		max	1	0·292	1·01	0·116	0·994	0·167	0·993	0·445	0·951	0·29
	log		1·29	0·265	1·57	0·117	0·745	0·226	0·851	0·544	0·503	0·166
		mean	1·05	0·249	1·04	0·378	0·966	0·366	0·99	0·65	0·809	0·236
	log		1·86	0·0669	1·91	0·0911	0·702	0·263	0·834	0·608	0·396	0·174
G-CSF (pg/ml)		max	1	0·262	1	0·0541	1	0·861	1	0·869	1	0·718
	log		1·48	0·179	3·48	**0·0244**	0·99	0·972	0·903	0·738	1·19	0·668
		mean	1	0·123	1	**0·0312**	1	0·762	0·999	0·64	1	0·89
	log		1·85	0·0888	3·36	**0·0431**	1·02	0·949	0·814	0·561	1·22	0·69
MCP-1 (pg/ml)		max	1	0·745	1	0·0589	1	0·704	0·999	0·213	1	0·108
	log		1·27	0·546	3·09	0·0571	0·936	0·874	0·628	0·328	2·81	0·132
		mean	1	0·876	1	0·27	1	0·81	0·995	0·159	1	0·0977
	log		1·22	0·634	1·64	0·351	1·01	0·981	0·541	0·252	2·69	0·156
MIP-1b (pg/ml)		max	0·998	0·551	1	0·451	0·999	0·699	0·997	0·339	0·99	0·165
	log		0·832	0·655	1·63	0·397	0·84	0·7	0·96	0·931	0·446	0·237
		mean	1	0·986	1·01	0·449	1	0·864	0·999	0·87	0·974	0·12
	log		1·04	0·938	1·7	0·435	1·04	0·936	1·13	0·833	0·231	0·126

EF = ejection fraction. E/e’ = ratio of E-wave to e’. avcIB = average calibrated integrated backscatter of septal and posterior LV wall. NEPI = norepinephrine. EPI = epinephrine. DOPA = dopamine. OR = odds ratio.

Supplemental table 2b: **Logistic regression of biomarkers during acute hospitalization**

**Long-term parameters**			**Linear regression**					
			EF		E/e'		avcIB	
			slope	p value	slope	p value	slope	p value
Cortisol (µg/24h, Urine)		max	-0·0102	0·426	-0·00346	0·427	-0·00165	0·855
	log		-2·19	0·326	-0·505	0·509	0·587	0·71
		mean	-0·00619	0·811	0·00161	0·855	0·0194	0·278
	log		-1·9	0·473	-0·0518	0·954	2·37	0·195
Cortisol (pg/ml, Serum)		max	-0·0767	0·115	-0·00433	0·869	0·0123	0·838
	log		-5·04	0·143	-0·0359	0·985	1·58	0·706
		mean	-0·0845	0·063	-0·00285	0·909	0·00434	0·939
	log		-6·49	**0·0257**	0·0632	0·969	0·405	0·914
NEPI (µg/24h, Urine)		max	-0·0158	0·578	-0·0088	0·409	-0·0254	0·303
	log		-1·69	0·237	-0·65	0·229	-1·04	0·408
		mean	-0·0407	0·611	-0·0266	0·377	-0·0348	0·62
	log		-1·85	0·289	-0·841	0·198	-0·719	0·641
EPI (µg/24h, Urine)		max	-0·0203	0·212	-0·00793	0·197	-0·0103	0·475
	log		-0·825	0·54	-1·03	0·0352	-0·979	0·405
		mean	-0·0695	0·33	-0·0319	0·233	-0·0461	0·465
	log		-0·933	0·6	-1·21	0·0621	-0·828	0·597
DOPA (µg/24h, Urine)		max	-0·00418	0·52	-0·00206	0·398	-0·00364	0·519
	log		-2·04	0·262	-0·972	0·153	-1·11	0·485
		mean	-0·00759	0·513	-0·00432	0·321	-0·00724	0·475
	log		-2·17	0·285	-0·994	0·192	-0·857	0·634
IL-1β (pg/ml)		max	-0·0182	0·102	0·00347	0·374	0·00627	0·504
	log		-1·09	0·274	0·265	0·441	0·567	0·49
		mean	-0·105	0·147	0·0222	0·378	0·0384	0·527
	log		-1·24	0·414	0·439	0·399	0·773	0·534
IL-2 (pg/ml)		max	0·0162	0·514	0·011	0·193	0·0449	**0·0148**
	log		0·555	0·618	0·362	0·341	2·03	**0·014**
		mean	0·0991	0·398	0·0515	0·197	0·201	**0·00127**

	log		1·6	0·318	0·534	0·332	3·06	**0·00547**
IL-4 (pg/ml)		max	0·106	0·0791	0·000732	0·973	0·0144	0·779
	log		1·56	0·275	0·191	0·7	1·39	0·229
		mean	0·175	0·0451	-0·0115	0·714	-0·0184	0·806
	log		2·62	0·121	0·00254	0·997	1·16	0·394
IL-5 (pg/ml)		max	-0·22	0·236	0·0472	0·464	0·0763	0·623
	log		-1·25	0·515	0·291	0·661	0·793	0·621
		mean	-0·351	0·634	0·112	0·657	0·149	0·807
	log		-1·87	0·526	0·425	0·676	1·05	0·667
IL-6 (pg/ml)		max	-0·0075	0·346	-0·00173	0·531	-0·00332	0·617
	log		-0·717	0·509	-0·23	0·538	0·443	0·622
		mean	0·000273	0·989	-0·00945	0·159	-0·0124	0·448
	log		-0·871	0·549	-0·379	0·448	0·542	0·649
IL-7 (pg/ml)		max	-0·066	0·17	0·014	0·402	0·0306	0·448
	log		-1·77	0·257	0·414	0·445	1·41	0·277
		mean	-0·192	0·293	0·0476	0·452	0·11	0·472
	log		-1·94	0·321	0·434	0·523	1·62	0·321
IL-8 (pg/ml)		max	-0·0108	0·312	0·00269	0·466	-0·0017	0·847
	log		-0·201	0·869	0·0498	0·905	-1·24	0·196
		mean	0·0392	0·424	0·000814	0·962	-0·0521	0·186
	log		0·481	0·745	0·0294	0·954	-1·95	0·0916
IL-10 (pg/ml)		max	0·00617	0·871	0·000891	0·946	0·0123	0·694
	log		0·517	0·662	0·275	0·498	1·18	0·232
		mean	0·13	0·413	-0·00388	0·944	0·039	0·768
	log		1·65	0·33	0·432	0·46	1·71	0·224
IL-12 (pg/ml)		max	-0·0299	0·56	0·00603	0·733	0·0366	0·386
	log		0·194	0·885	-0·03	0·948	1·12	0·322
		mean	-0·00904	0·972	0·0259	0·767	0·125	0·554
	log		0·825	0·688	0·0393	0·956	1·4	0·417
IL-13 (pg/ml)		max	0·204	0·147	-0·00994	0·84	-0·0573	0·628
	log		0·565	0·769	0·2	0·762	-0·474	0·764
		mean	0·267	0·0774	-0·016	0·766	-0·0738	0·565

	log		1·82	0·42	0·347	0·656	-0·711	0·702
IL-17 (pg/ml)		max	0·00617	0·871	0·000891	0·946	0·0123	0·694
	log		0·517	0·662	0·275	0·498	1·18	0·232
		mean	0·13	0·413	-0·00388	0·944	0·039	0·768
	log		1·65	0·33	0·432	0·46	1·71	0·224
GM-CSF (pg/ml)		max	-0·00523	0·73	0·00684	0·182	0·0266	**0·024**
	log		-0·7	0·575	0·602	0·154	2·77	**0·00503**
		mean	0·0152	0·872	0·0436	0·172	0·192	**0·000597**
	log		0·218	0·9	0·872	0·135	3·6	**0·00516**
INFγ (pg/ml)		max	-0·0000421	0·998	0·00516	0·295	0·00757	0·526
	log		0·129	0·897	0·153	0·653	0·386	0·637
		mean	0·0367	0·487	0·0179	0·323	0·037	0·395
	log		0·679	0·619	0·267	0·57	0·788	0·482
TNFα (pg/ml)		max	-0·0234	0·151	0·00468	0·41	0·00948	0·488
	log		-0·852	0·456	0·365	0·352	0·634	0·496
		mean	-0·103	0·265	0·0268	0·403	0·0591	0·443
	log		-0·494	0·761	0·564	0·308	0·813	0·538
G-CSF (pg/ml)		max	-0·00651	0·433	0·00154	0·592	0·00231	0·737
	log		0·315	0·79	-0·227	0·575	-0·101	0·917
		mean	0·0225	0·581	0·00263	0·851	-0·00114	0·973
	log		1·61	0·288	-0·469	0·369	-0·626	0·621
MCP-1 (pg/ml)		max	-0·00359	0·486	0·000628	0·724	0·0000448	0·992
	log		-1·06	0·598	0·266	0·701	-1·15	0·476
		mean	0·000331	0·958	-0·0000298	0·989	-0·00194	0·704
	log		-0·0474	0·98	0·117	0·854	-1·47	0·325
MIP-1b (pg/ml)		max	-0·0185	0·267	-0·00234	0·686	-0·00963	0·487
	log		-2·24	0·416	-0·364	0·703	-1·23	0·59
		mean	-0·0232	0·527	-0·00247	0·845	-0·0302	0·312
	log		-1·71	0·638	-0·346	0·782	-2·53	0·391

EF = ejection fraction. E/e' = ratio of E-wave to e'. avcIB = average calibrated integrated backscatter of septal and posterior LV wall. NEPI = norepinephrine. EPI = epinephrine. DOPA = dopamine. OR = odds ratio.

Supplemental table 3a: **Linear regression of biomarkers between discharge from acute hospitalization and study date**

Long-term parameters			Logistic regression									
			EF < 50%		EF < 40%		E/e' > 8		E/e' ≥ 12		avcIB > -15	
			OR	p value	OR	p value	OR	p value	OR	p value	OR	p value
Cortisol (µg/24h, Urine)		max	1	0·444	1	0·224	0·998	0·414	0·997	0·359	0·999	0·84
	log		1·34	0·55	1·43	0·55	0·546	0·368	0·688	0·436	1·05	0·942
		mean	1	0·695	1	0·431	0·996	0·434	0·999	0·882	1	0·619
	log		1·37	0·576	1·32	0·667	0·619	0·482	0·785	0·67	2·08	0·408
Cortisol (pg/ml, Serum)		max	1·04	0·21	1·02	0·204	0·986	0·342	1	0·918	0·983	0·559
	log		5·66	0·148	11	0·151	0·343	0·28	1·42	0·697	0·573	0·639
		mean	1·14	0·288	1·02	0·189	0·989	0·385	1	0·972	0·971	0·576
	log		38·8	0·173	8·75	0·147	0·481	0·372	0·984	0·984	0·41	0·519
NEPI (µg/24h, Urine)		max	0·999	0·847	0·998	0·778	0·993	0·284	0·999	0·895	1	0·961
	log		1·32	0·413	1·2	0·665	0·707	0·348	0·941	0·863	1·45	0·428
		mean	0·994	0·729	0·996	0·863	0·988	0·518	0·997	0·887	1·01	0·514
	log		1·28	0·539	1·27	0·639	0·698	0·416	0·864	0·733	2	0·273
EPI (µg/24h, Urine)		max	1	0·371	1·01	0·221	0·996	0·32	0·978	0·266	0·994	0·653
	log		1·17	0·604	1·41	0·383	0·827	0·559	0·547	0·123	1·12	0·776
		mean	1·02	0·395	1·03	0·165	0·987	0·44	0·962	0·345	0·994	0·817
	log		1·21	0·632	1·5	0·436	0·889	0·781	0·506	0·169	1·46	0·498
DOPA (µg/24h, Urine)		max	1	0·572	1	0·58	0·999	0·473	0·999	0·72	1	0·919
	log		1·43	0·426	1·83	0·343	0·62	0·346	0·651	0·337	1·4	0·588
		mean	1	0·602	1	0·388	0·998	0·381	0·998	0·533	1	0·996
	log		1·4	0·487	2·1	0·294	0·636	0·402	0·612	0·33	1·49	0·561
IL-1β (pg/ml)		max	1·01	0·426	1·01	0·319	1·09	0·303	1·01	0·328	0·986	0·566
	log		1·1	0·654	1·45	0·128	1·62	0·116	1·49	0·103	0·892	0·721
		mean	1·03	0·451	1·04	0·298	1·35	0·318	1·06	0·297	0·907	0·524
	log		1·16	0·645	1·65	0·159	2·81	0·106	1·87	0·0914	0·726	0·573
IL-2 (pg/ml)		max	0·994	0·376	0·999	0·858	1·01	0·282	1·02	0·0787	1	0·488
	log		0·872	0·56	0·994	0·984	1·42	0·211	1·75	0·0576	1·26	0·437
		mean	0·971	0·369	0·993	0·825	1·12	0·247	1·08	0·109	1·03	0·176

	log		0·778	0·465	0·821	0·65	1·82	0·168	2·1	0·0732	1·45	0·338
IL-4 (pg/ml)		max	0·992	0·557	0·973	0·584	1·05	0·441	1	0·746	1·01	0·504
	log		0·892	0·704	0·763	0·522	1·71	0·187	1·4	0·317	1·61	0·207
		mean	0·975	0·469	0·785	0·409	1·08	0·572	0·984	0·697	1	0·99
	log		0·868	0·698	0·502	0·36	1·74	0·292	1·07	0·876	1·55	0·283
IL-5 (pg/ml)		max	1·04	0·473	1·1	0·231	1·32	0·129	1·08	0·218	0·888	0·528
	log		1·05	0·901	1·98	0·163	2·18	0·137	1·76	0·233	0·698	0·577
		mean	1·09	0·608	1·29	0·139	2·18	0·184	1·19	0·287	0·731	0·53
	log		1·35	0·628	2·93	0·135	4·2	0·123	1·93	0·341	0·498	0·522
IL-6 (pg/ml)		max	1	0·8	1	0·501	0·998	0·295	1	0·242	0·997	0·478
	log		0·888	0·603	1·11	0·691	0·864	0·532	1·41	0·221	0·99	0·974
		mean	0·995	0·374	0·998	0·791	0·992	0·253	1	0·901	0·995	0·666
	log		0·866	0·638	1·06	0·88	0·821	0·531	1·45	0·306	1·07	0·875
IL-7 (pg/ml)		max	1·01	0·544	1·02	0·162	1·03	0·193	1·02	0·175	0·97	0·42
	log		1·07	0·842	1·84	0·194	1·7	0·155	1·8	0·205	0·875	0·771
		mean	1·01	0·711	1·07	0·139	1·09	0·104	1·06	0·227	0·911	0·264
	log		1·08	0·848	1·91	0·261	1·97	0·137	1·71	0·332	0·711	0·549
IL-8 (pg/ml)		max	1	0·59	1	0·238	1	0·507	1	0·133	0·985	0·221
	log		0·931	0·778	1·29	0·446	1·11	0·682	1·08	0·796	0·561	0·129
		mean	0·995	0·643	1	0·839	1	0·679	1·01	0·633	0·971	0·209
	log		0·906	0·75	1·11	0·786	1·16	0·64	0·974	0·942	0·496	0·123
IL-10 (pg/ml)		max	0·994	0·491	1·01	0·447	0·994	0·435	1·01	0·493	0·985	0·544
	log		0·752	0·27	1·32	0·351	1·01	0·963	1·36	0·303	1·09	0·798
		mean	0·961	0·326	1	0·995	0·961	0·281	1·01	0·724	0·971	0·664
	log		0·673	0·293	1·13	0·767	0·975	0·945	1·5	0·323	1·21	0·699
IL-12 (pg/ml)		max	0·998	0·858	1·01	0·3	1·01	0·61	1·01	0·229	0·977	0·579
	log		0·735	0·294	1·1	0·766	1·08	0·803	1·38	0·344	1·03	0·942
		mean	0·988	0·814	1·06	0·34	1·05	0·49	1·06	0·278	0·89	0·515
	log		0·717	0·45	1·12	0·817	1·34	0·523	1·38	0·519	0·876	0·833
IL-13 (pg/ml)		max	0·974	0·48	0·994	0·884	1·09	0·396	0·997	0·942	0·962	0·681
	log		0·927	0·85	1·2	0·697	1·8	0·238	1·29	0·574	0·687	0·566
		mean	0·962	0·474	0·964	0·685	2·39	0·158	0·96	0·687	0·779	0·635

	log		0·936	0·889	0·988	0·983	7·21	0·102	0·991	0·987	0·514	0·546
IL-17 (pg/ml)		max	0·994	0·491	1·01	0·447	0·994	0·435	1·01	0·493	0·985	0·544
	log		0·752	0·27	1·32	0·351	1·01	0·963	1·36	0·303	1·09	0·798
		mean	0·961	0·326	1	0·995	0·961	0·281	1·01	0·724	0·971	0·664
	log		0·673	0·293	1·13	0·767	0·975	0·945	1·5	0·323	1·21	0·699
GM-CSF (pg/ml)		max	1	0·46	0·999	0·869	1·02	0·218	1·03	0·0984	1·01	0·254
	log		1·17	0·561	1·34	0·381	1·67	0·104	2·04	0·0925	2·05	0·131
		mean	1·01	0·526	0·972	0·509	1·04	0·344	1·04	0·165	1·05	**0·0424**
	log		1·22	0·589	0·981	0·965	1·98	0·119	2	0·163	2·65	0·0907
INFγ (pg/ml)		max	0·997	0·435	1	0·465	1	0·413	1·01	0·0702	0·997	0·587
	log		0·801	0·302	1·09	0·72	1·17	0·478	1·38	0·254	0·997	0·993
		mean	0·982	0·252	1	0·932	1·01	0·42	1·02	0·109	0·985	0·577
	log		0·716	0·267	1·01	0·974	1·33	0·348	1·52	0·262	0·954	0·906
TNFα (pg/ml)		max	1	0·487	1·01	0·298	1·01	0·536	1·01	0·382	0·986	0·554
	log		1·02	0·944	1·34	0·318	1·24	0·395	1·7	0·112	0·843	0·618
		mean	1·01	0·574	1·03	0·227	1·06	0·382	1·04	0·219	0·855	0·266
	log		0·993	0·984	1·25	0·58	1·53	0·261	1·81	0·184	0·619	0·349
G-CSF (pg/ml)		max	1	0·875	1	0·224	1	0·837	1	0·184	0·998	0·598
	log		0·693	0·197	1·13	0·701	0·946	0·829	1·18	0·611	1·15	0·712
		mean	0·989	0·262	1	0·826	0·996	0·661	1·01	0·249	0·987	0·476
	log		0·517	0·113	0·832	0·613	0·828	0·586	1·04	0·923	1·02	0·957
MCP-1 (pg/ml)		max	1	0·448	1	0·109	1	0·432	1	0·831	1	0·846
	log		1·38	0·447	2·31	0·175	1·36	0·487	0·806	0·667	0·449	0·199
		mean	1	0·619	1	0·426	1	0·726	0·997	0·327	1	0·474
	log		1·37	0·425	1·39	0·485	1·18	0·683	0·607	0·314	0·519	0·282
MIP-1b (pg/ml)		max	0·998	0·575	1·01	0·192	0·998	0·591	1	0·91	0·981	0·139
	log		0·628	0·433	2·02	0·345	0·82	0·741	1·19	0·8	0·241	0·131
		mean	0·993	0·385	1·01	0·488	0·999	0·873	1	0·803	0·956	0·0863
	log		0·471	0·338	1·47	0·678	0·903	0·896	1·17	0·866	0·0819	0·0666

EF = ejection fraction. E/e’ = ratio of E-wave to e’. avcIB = average calibrated integrated backscatter of septal and posterior LV wall. NEPI = norepinephrine. EPI = epinephrine. DOPA = dopamine. OR = odds ratio.

Supplemental table 3b: **Logistic regression of biomarkers between discharge from acute hospitalization and study date**
